# Metal Nanoparticles/Porous Silicon Microcavity Enhanced Surface Plasmon Resonance Fluorescence for the Detection of DNA

**DOI:** 10.3390/s18020661

**Published:** 2018-02-23

**Authors:** Jiajia Wang, Zhenhong Jia

**Affiliations:** 1 School of Chemistry and Chemical Engineering, Xinjiang University, Urumqi 830046, China; wjiajia_1127@163.com; 2 School of Information Science and Engineering, Xinjiang University, Urumqi 830046, China

**Keywords:** porous silicon microcavity, gold nanoparticles, DNA, fluorescence

## Abstract

A porous silicon microcavity (PSiMC) with resonant peak wavelength of 635 nm was fabricated by electrochemical etching. Metal nanoparticles (NPs)/PSiMC enhanced fluorescence substrates were prepared by the electrostatic adherence of Au NPs that were distributed in PSiMC. The Au NPs/PSiMC device was used to characterize the target DNA immobilization and hybridization with its complementary DNA sequences marked with Rhodamine red (RRA). Fluorescence enhancement was observed on the Au NPs/PSiMC device substrate; and the minimum detection concentration of DNA ran up to 10 pM. The surface plasmon resonance (SPR) of the MC substrate; which is so well-positioned to improve fluorescence enhancement rather the fluorescence enhancement of the high reflection band of the Bragg reflector; would welcome such a highly sensitive in biosensor.

## 1. Introduction

PSi is a biocompatible photonic material, which has potential applications in the field of biosensing [1,2]. In particular, functionalized PSi can be fabricated into multiple sensors. Rapid desorption of nicotine from respiratory gas has been achieved by analytical ionization on the porous silicon surface [3]. Metal (Ni and Bi) coatings on the PSi nanostructure surface were prepared for high capacity and the stable performance anode material lithium ion battery [4]. As previously described, PSi has been used for the preparation of a variety of sensors, especially in the field of optical sensor.

Currently, labeled-free and labeled biosensors are summarized as the two common types of PSi optical biosensors. The variation of the effective refractive index of the PSi film is caused by the entrance of biomolecules attached to the PSi, which is directly translated to the movement of the spectrum [5,6,7] in labeled-free biosensor, and the change of luminescence center [8,9]. Labeled biological detection has the characteristics of high sensitivity, good selectivity, less biomass, and fast response time. Two main approaches utilizing fluorophore-labeled for the amplification of the optical signal in biosensor applications were developed. In refractive index-enhanced reflectance spectrum movement, the binding of fluorophore-labeled molecules to biomolecular recognition elements attached to a PSi sensor surface is probed by the movement of reflectance spectrum. This method was implemented by using the high refractive index of quantum dots [10]. In SPR-enhanced fluorescence, the enhanced field of metal NPs increases the fluorescence intensity of the fluorophore-labeled molecules [11,12,13].

Metal NPs combined with photonic crystal technology can further enhance the optical signal and improve sensitivity. Fluorescence enhancement of the Au NPs in TiO_2_ inverse opal photonic crystal has achieved SARS and HIV virus detection using label-free DNA, the signal intensity and the detection sensitivity is improved by one order of magnitude [14]. Park et al. [15] reported the formation of photonic crystals from the resin and further enhanced the fluorescence by embedding Ag NPs. In order to further explore the new method of high sensitivity detection of the biology based on the PSi photonic devices, the combination of the unique photon transmission control capability (band-gap of Bragg reflector, defect mode of MC etc.) of the PSi photonic crystal device noble metal NPs is dedicated to develop a biosensor with better performance based on fluorescence detection. In recent years, some new advances in the fluorescence enhancement analysis of the probe molecules on the PSi Bragg mirror obtained by our group are summarized. When the fluorescence emission peak of the probe molecules falls into the high reflection band of PSi Bragg reflector, the fluorescence intensity of the probe can be increased [16]; DNA has been specifically selected by fluorescence resonance energy transfer(FRET) from quantum dots and Au NPs on PSi Bragg reflector substrate [17]. However, few studies are concerned with the PSi photonic crystal fluorescence enhancement for biological detection [18,19,20]. Especially, the combination of metal NPs and the PSi photonic crystal is used to further improve the performance of biosensors based on fluorescence detection technology. Therefore, the PSi with biological characteristics, large surface area characteristics, and easy preparation of all kinds of photonic devices could be combined with SPR to futher enhance the sensitivity of biosensors for detecting fluorescence changes.

**Figure 1:**
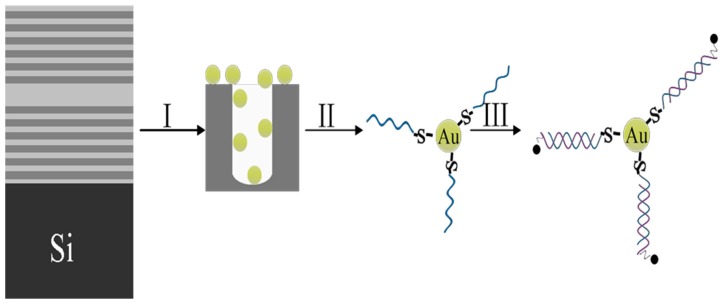
The flow-process diagram of DNA detection on metal NPs /PSiMC device. (I) Au NPs adsorbed on functionalized PSiMC; (II) the immobilization of target DNA on Au NPs /PSiMC device substrate; (III) the hybridization of probe RRA-DNA sequences with target DNA.

This paper mainly studies (1) the impact of Au NPs adsorbed on PSiMC and the Bragg reflector on the fluorescence enhancement, (2) the specificity identification of DNA on the Au NPs/PSiMC device sensor, and (3) the linear correlation between fluorescence intensity and the concentrations of RRA-DNA. The flow chart of the PSiMC device substrate for the detection of DNA is shown in Figure 1. Firstly, the PSiMC were functionalized to adsorb Au NPs; secondly, the thiol modified DNA was connected with the Au NPs as a target DNA; then, complementary DNA modified with RRA was hybridized with target DNA. 

## 2. Experimental Details

### 2.1. Fabrication of PSiMC

**Figure 2:**
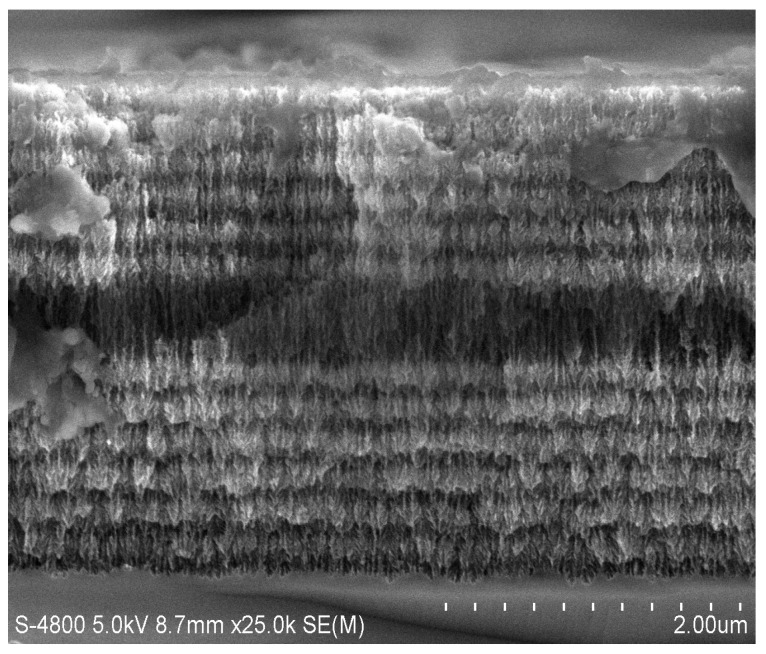
The cross-section image of PSiMC.

PSi was prepared by standard anodic etching technique [21]. The material used is p-type monocrystal silicon <100> (resistivity 0.03–0.06 Ω·cm). The etching solution for electrochemical etching of PSi is 10% hydrofluoric acid alcohol solution. Prior to preparation of the samples, the parasitic layer of highly doped p-type silicon wafer was removed by short electrochemical etching and dissolution in NaOH. It has been proved that the existence of this parasitic film affects the optical properties of the photonic structure, reduces the pore size of the surface [22], and prevents the molecules from entering the porous silicon structure. The preparation parameters of PSiMC are shown in Table 1. As shown in Figure 2, the distribution of MC layers: a cavity layer is introduced into the two symmetrically distributed Bragg reflectors; the corrosion current density of cavity layer is 110 mA/cm^2^, the A layer is a high porosity layer with a current density of 110 mA/cm^2^, the B layer is the low porosity layer, and the current density is 60 mA/cm^2^. The arrangement of the PSiMC structure is (AB)_6_A_4_(BA)_6_, and the resonance cavity wavelength is 635 nm. Bragg reflectors with 14 cycles, which are arranged as (AB)_14_, are predicted to have the same thickness of MC. The fresh samples were thoroughly cleaned with deionized water and dried under N_2_ airsteam.

**Figure 3:**
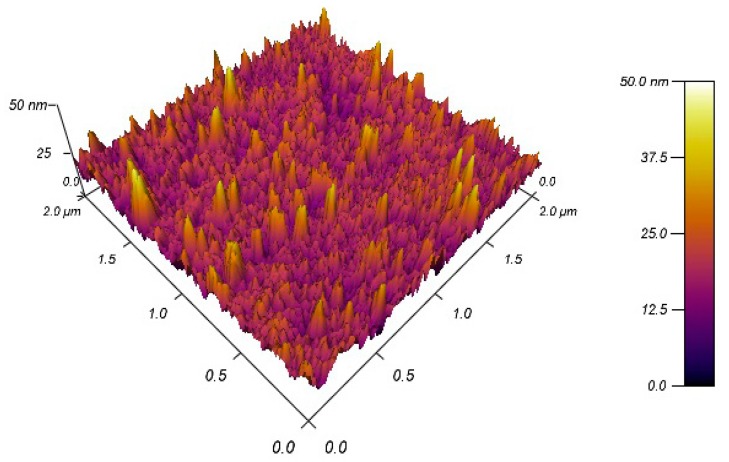
AFM image of PSi layer.

More significantly, however, the 3D AFM image corresponding to the porous silicon layer, was illustrated in Figure 3. In fact, AFM images have shown, before gold deposition, almost irregular and randomly dis-tributed nano-crystalline silicon pillars (pointed silicon tip). Consequently, the gold deposit will follow this morphology and it will overlay each surface at a certain distance.

### 2.2. The Functionalization of PSi

The fresh PSi devices are easy to oxidize, which affects the stability of the fabricated sensors. The fresh PSi devices are fully oxidized in hydrogen peroxide (30%) for 24 h at room temperature. After oxidation, the PSi device surface will form relatively stable silicon oxide, and then, oxidized PSi samples are aminated for 1h in 5% (3-aminopropyl) triethoxysilane (APTES), cleaned with deionized water and dried under N_2_ airsteam. The surface of the PSi after aminated treatment is positively charged and can electrostatically adsorb Au NPs prepared by citrate reduction [23,24]. The interaction was stable and irreversible.

### 2.3. The Fabrication of Gold Nanoparticles

Au NPs were prepared by hydrothermal method given in [25]. Chlorauric acid solution (50 mL, 1.0 mM) was heated to 100 ℃ by heat collecting magnetic heating stirrer. Under boiling, sodium citrate solution was quickly added (8 mL, 1 wt %), with continuous heating while stirring. Until the color of the solution changed from yellow to red wine, heating did not commence, and cooling to room temperature was preserved. After being soaked in colloidal Au solution for 5 h, the aminated PSi samples were removed and rinsed with deionized water. 

**Figure 4:**
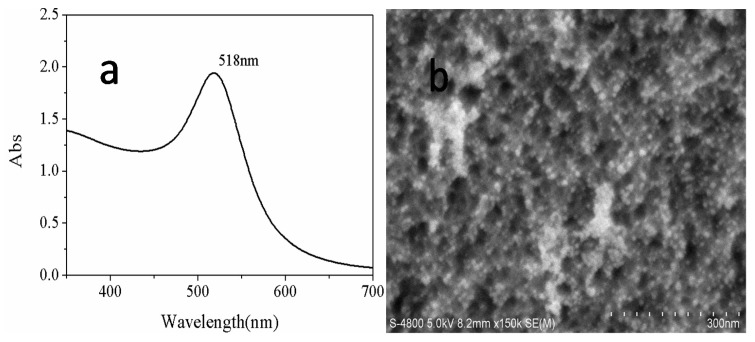
(**a**) The UV-Vis spectra of Au NPs, and (**b**) surface morphology of PSiMC after deposition of Au NPs.

The absorption spectra of the Au NPs are presented in Figure 4a, and the strongest absorption peak is located at 518 nm. Regarding the morphology of functionalized PSi deposited with Au NPs as shown in Figure 4b, Au NPs evenly distributed on the surface and the pores of PSi can be clearly observed, and the diameter of Au NPs is 7 ± 2 nm. A zoom-in SEM characterization figure of porous silicon microcavity (PSiMC) and the hybrid nanostructure are provided in Figure S1 (supporting information).

### 2.4. DNA Fragment linked to Metal NPs

DNA fragments are purchased from the INVITROGEN TRADING Co., Ltd. (Shanghai, China). The sequence of DNA fragments is shown in Table 2; sample 1 and sample 2 are applicable for Au NPs/PSiMC metal enhancement substrate; sample 3 is applied to bare PSiMC substrate without Au NPs.

**Figure 5:**
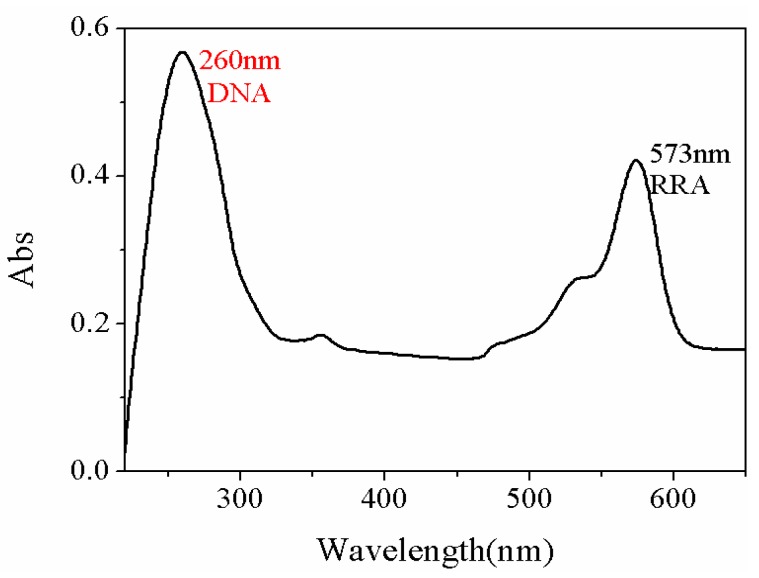
The UV-Vis absorption spectra of RRA-DNA.

TE buffer is made up of Tris(hydroxymethyl)aminomethane (Tris) and ethylenediaminetetraacetic acid (EDTA), which is mainly used to dissolve DNA and can store DNA steadily. Before being diluted with TE buffer (pH = 8.0), the DNA fragments were centrifuged for 30 s and aggregated to the bottom of the tube. In aseptic operation, DNA fragments were diluted to 10 µM, stored at −20 °C, and protected from light. The DNA primer modified with 50 µL Thiol-C6 S-S (THS) was activated with Tris (2-carboxyethyl) phosphine hydrochloride (TCEP, 1 mM) to cut off the disulfide bond for 1 h at room temperature. The concentration of TCEP is 1 mM. 50 µL THS-DNA (10 µM) was dripped into PSiMC adsorbed with Au NPs, incubated for 10 h in a 37 °C incubator and removed from the unconnected DNA with TE buffer. Then, the samples were blocked with EA (3 M HEPES buffer, pH = 9.0) 37 °C for 1h. THS-DNA was immobilized on Au NPs as a target DNA, and the final aim was to achieve hybridization with the captured probe RRA-DNA. The UV-Vis absorption spectrum of RRA-DNA is shown in Figure 5. The peak at 260 nm is the absorption peak of DNA, and the absorption peak of RRA is located at 572 nm. 40 µL Rhodamine Red o-X (RRA)-modified DNA fragments with a concentration of 10 µM–10^−4^ µM were dripped on PSiMC linked with target DNA, and incubated at 37 °C for 10 h. Then, the sensors were cleaned with TE buffer and dried.

### 2.5. Measurements

The measurement of fluorescence spectrum employed the UV-Vis fluorescence spectrophotometer (Hitachi F-4600, Tokyo, Japan) with a slit width of 5 nm. The excitation power was 700 mW, and the response time was 0.004 s. The data were collected for five different positions from each sample, and averaged.

The reflectance spectra were collected using U-4100 (Hitachi, Tokyo, Japan). The incident angle is 5°. 

Measurement of surface topography was achieved by FESEM (ZEISS SUPRA55 VP, Oberkochen, Germany).

## 3. Results and Discussion

**Figure 6:**
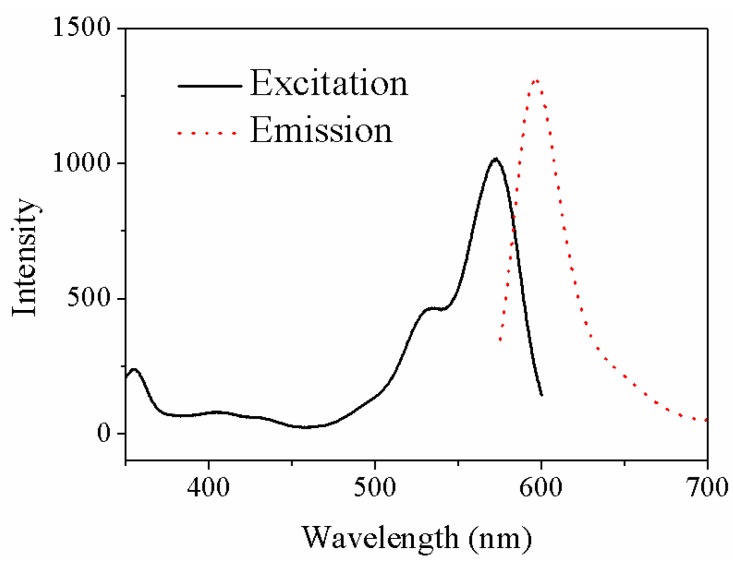
The absorption spectrum and emission spectrum of RRA-DNA fragment.

The excitation and emission fluorescence spectra of RRA-DNA fragment are described in Figure 6. The main excitation peaks are 360 nm, 530 nm and 572 nm, respectively. The emission wavelength is 597 nm, which is close to the fluorescence excitation wavelength (572 nm) of RRA-DNA; when measured, it is not conducive to obtaining the complete emission fluorescence spectra. The ultraviolet light of 360 nm easily causes damage to biological molecules, so the excitation wavelength of 530 nm is adopted in the experiment.

**Figure 7:**
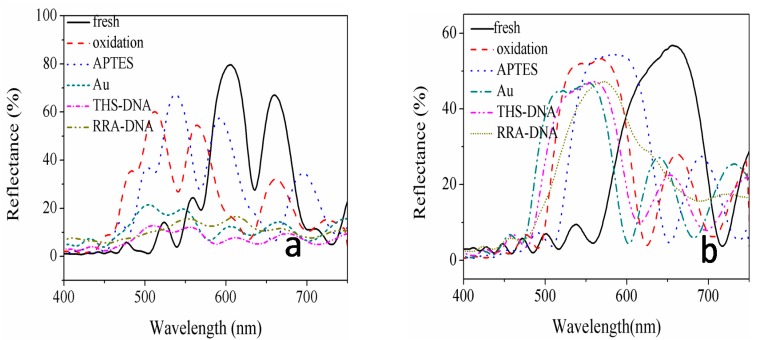
The reflection spectra vary with the preparation process of (**a**) PSiMC biosensor, and (**b**) Bragg reflector sensor.

The changes in the reflectance spectra of the PSiMC and PSi Bragg reflector substrate prepared for biosensor are presented in Figure 7. In the process of sensor preparation, the resonant peak and band-gap center of the two devices are shifting with every process. Finally, the resonant peak and band-gap center after the detection of RRA-DNA are expected to coincide with the fluorescence emission peak of RRA-DNA detected on the sensors, through the reasonable choice of the wavelength for the resonant peak and band-gap center of the two devices is 635 nm. The whole process of experimentation involved the following: the fabrication of fresh PSi, oxidation, alkylation, adsorption of Au NPs, linked target THS-DNA, and detection probe RRA-DNA. Blue shift of the reflection spectrum reached 100 nm from the complete oxidation process; red shift of the reflection spectrum by alkylating is 30 nm; deposition of Au NPs gave rise to the blue shift of reflection spectrum; the resonant cavity wavelength of the PSiMC and the central wavelength of the band-gap of PSi Bragg reflector are both at 530 nm; both THS-DNA connection and RAA-DNA detection cause the red shift of the reflection spectra. After the connection of RAA-DNA to the substrates, the resonance peak wavelength and the center band-gap wavelength shifted to 585 nm. 

**Figure 8:**
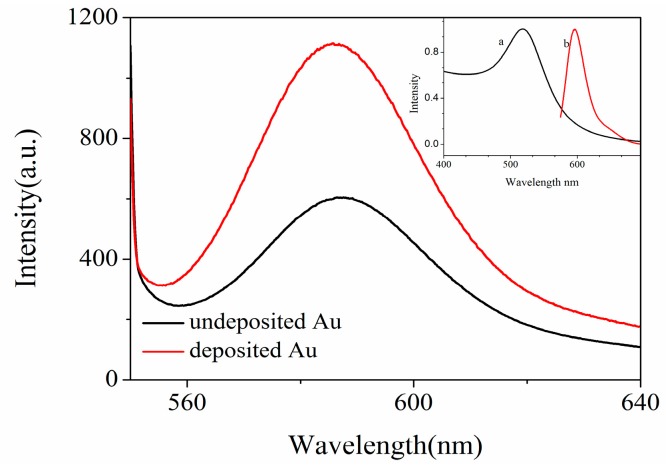
The fluorescence spectra of RRA-DNA obtained on Au NPs/PSiMC sensor substrate and PSiMC substrate without Au NPs (the concentration of RRA-DNA is 1 µM). The inset map shows (**a**) the absorption spectrum of Au NPs and (**b**) the emission spectrum of RRA.

**Figure 9:**
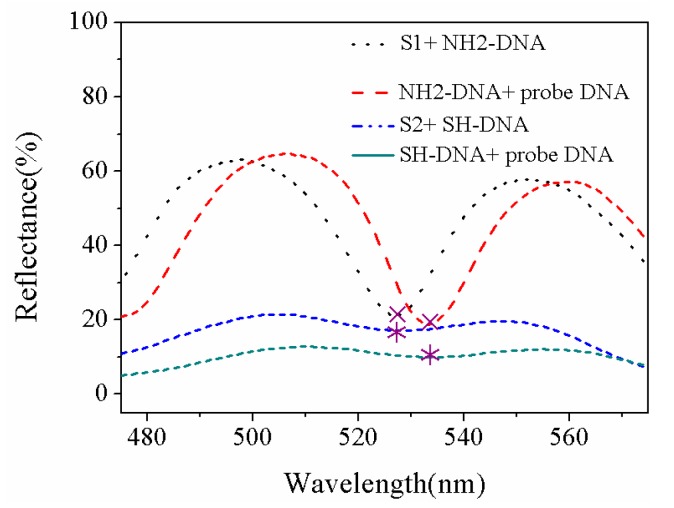
The movement of the reflection spectra from the probe DNA binds to the sensor substrates. The first type sensor substrate (S1) functional with glutaraldehyde, is easy to connect with amino-modified DNA fragments; the second type sensor substrate (S2) is readily connected sulfhydryl -modified DNA by adsorbed Au NPs.

Two kinds of PSiMC sensors were fabricated. One is a fluorescence enhancement sensor based on the surface of Au NPs/PSi substrate; THS-DNA fragment of sample 1 in Table 2 is used as a target DNA. The other is a bare PSi substrate functionalized by glutaraldehyde without Au NPs, and target DNA is the NH_2_-DNA fragment in Table 2. The two sets of target DNA have the same base sequence, and the probe RRA-DNA fragment is complementary to the target DNA. The average intensity of the fluorescence emission peak on the two kinds of sensors substrate (five samples for every kind) is presented in Figure 8, enhanced fluorescence signals are observed on Au NPs/PSi substrates. To be far from the differences in fluorescence signal caused by the amount of probe DNA (complementary DNA) attached to each sensor, the excessive target DNA (50 µL) is required to attach to each sensor substrate, while 40 µL of fluorescently labeled complementary DNA was attached to each sensor. In this case, even if the target DNA attached to each substrate is different, ultimately, it does not affect the amount of specific binding of the probe DNA, considering that the enhancement of fluorescence signal is most likely due to the difference in the amount of probe DNA that they bind to each type of sensor. The movement of the reflection spectra from the probe DNA binds to each type of sensor exhibited in Figure 9. An almost identical movement occurred after complementary DNA detected on S1 (the first type sensor substrate) and S2 (the second type sensor substrate), hence two types of sensor substrates bind the same amount of probe DNA. Consequently, fluorescence enhancement for the two type sensors is independent of the different amount of target DNA. Generally, FRET occurs between Au NPs donor and the fluorescent receptor. The overlapping reduction between the emission spectrum of RRA and the absorption spectrum of Au NPs (as shown in inset map of Figure 8) is expected to keep away from the occurrence of FRET. PSi field is helpful to effectively regulate the distribution of Au NPs, which can reduce fluorescence quenching due to the tiny distance. The pore size of porous silicon is in 20–25 nm as presented in Figure S1 (supporting information), and the pores and silicon walls of PSi can help to regulate the distribution of Au NPs, so that the distance between Au NPs is not too dense. As shown in Figure S1, the distance between the Au NPs is 10–20 nm. In the system we fabricated, the distance between the Au NPs and the fluorophore immobilized on Au NPs was less than 5 nm (for 14 base pairs), resulting in fluorescence quenching [26]. However, the diameter of our Au NPs is 7 ± 2 nm, the distance between the Au NP surface and the fluorophore is about 4.5 nm, and the detected fluorescent enhancement in the presence of Au NPs is a factor of ~2. This moderate fluorescent enhancement value observed in our experiment is in line with the theoretical and experimental results obtained by Kang et al. [27]. They reported that when the diameter of Au NPs is 8 nm, and the distances between dye molecules and Au NPs is 4.5 nm, thus, the relative maximum fluorescent enhancement obtained is about 2. Then, it is plausible that the observed moderate plasmonic fluorescent enhancement effect can be due to single Au NP in our sensor. In the competition between fluorescence quenching and fluorescence enhancement, fluorescence enhancement is more advantageous. More significantly, however, the fluorophores connected to Au NPs deposited on PSi through DNA fragments act as an optical antenna [28]. According to the description in the article, the fluorescence enhancement starts to drop for very short distances, indicative for the onset of quenching. However, for fluorophores placed at such short distances from the surface of the Au NPs (3~5 nm), there still exists fluorescence enhancement. Fluorescence enhancement is more competitive than fluorescence quenching, which is a good illustration of fluorescence enhancement based on the fabricated Au NPs/PSi substrates.

**Figure 10:**
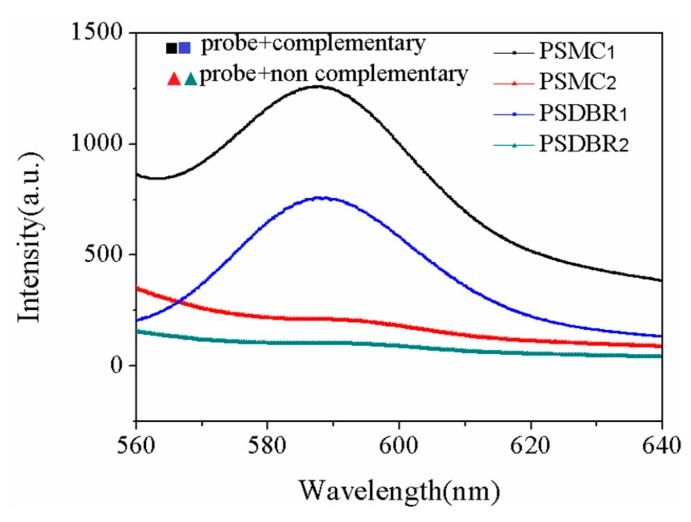
The fluorescence spectra of RRA-DNA detected on PSi biosensor. The concentration of RRA-DNA is 1 µM. There are two spectra from two different samples for one kind of sensor. One of the spectra is the fluorescence spectrum for the detection of complementary probe DNA (the solid square), and the other one is the spectrum for the detection of non complementary probe DNA (the solid regular triangle).

Au NPs were deposited on PSiMC for DNA detection; the fluorescence spectra are shown in Figure 10. THS-DNA fragment (THS-5’-GGCCTATCAGCTTG-3’) is arranged for the target DNA; two kinds probe DNA for PSiMC structure device have been chosen, which are complementary RRA-DNA (RRA-5’-CAAGCTGATAGGCC-3’) and non-complementary RRA-DNA (RRA-5’-GGCCTATCAGCTTG-3’). The target DNA is hybridized with the complementary RRA-DNA, and the DNA is detected by the fluorescence signal of RRA. Strong fluorescence signals can be observed from Figure 10. The non-complementary DNA cannot specifically be hybridized with target DNA, and fluorescent signals cannot be observed on the PSi sensor substrate. The enhanced fluorescence signal from Au NPs/PSiMC substrate is stronger than that obtained on Au NPs/PSi Bragg reflector. The fluorescence molecules are bound in the active layer of the PSiMC, which makes the interaction between the molecules and the optical field strong, and enhances the fluorescence emission of RRA. A similar effect has been reported, that is, the dye molecules (Rhodamine) in the PSiMC structure are expected to amplify the emission spectrum by optical excitation limited [29]. In addition, glucose oxidase (GOX) and fluorescein isothiocyanate (FITC) labeled streptavidin were confined in PSiMC, and the enhancement of fluorescence was demonstrated by comparing the surface fluorescence intensity [30]. In order to exclude the contingency of the experiment, five samples were prepared for each type with the same condition, five different points were collected for every sample, and the average value was taken. Although the fluorescence emission wavelength range in the high reflection band-gap of Bragg reflector structure (585 nm, Figure 7b), after the RRA-DNA is connected to the sensor substrate, can improve the detection of the fluorescent signal, the fluorescence enhancement on Au NPs/PSi substrate depends not only on the PSi photonic crystal structure, but also the plasma resonance with Au NPs. RRA-DNA is connected to the PSiMC, and the wavelength of the MC resonance peak is located at 585 nm. This occurred because of the fluorescence peaks overlap; therefore, it cannot achieve high fluorescence enhancement from high reflection, and the phenomenon of fluorescence enhancement can be interpreted as the plasma resonance produced by the combination of MC structure and Au NPs. Both the wavelengths of resonant cavity and band-gap are at 530 nm (fluorescence excitation wavelength) for PSi adsorbed with Au NPs as shown in Figure 7. Therefore, a strong SPR can be produced on the MC substrate. The advantage of fluorescence enhancement produced by SPR is more prominent than that caused by the reflection band-gap. 

Unlike that reported in this literature [29], spectral narrowing on PSiMC substrate for the fluorescence detection of RRA in this paper is not observed compared with the Bragg substrate. The reason for this phenomenon is that there are many layers of Bragg reflector in PSiMC, and the pore size of the PSiMC is not enough to allow the RRA-DNA molecules to enter into the deep layer of Bragg until entering the cavity layer.

**Figure 11:**
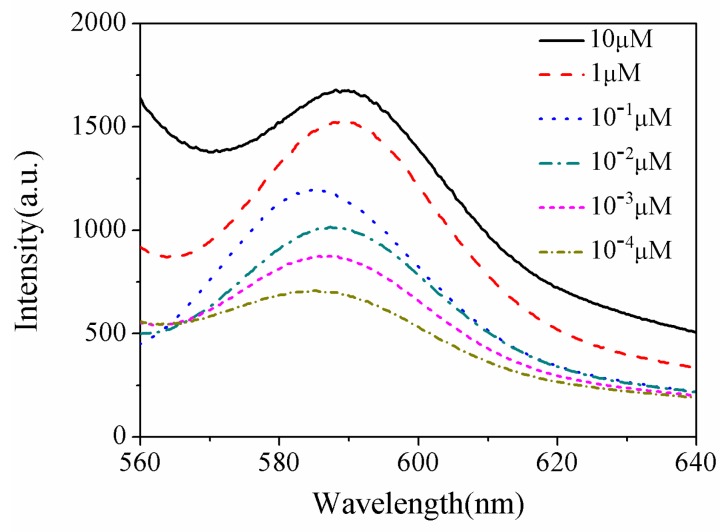
The fluorescence spectra of RRA-DNA with varying concentrations.

**Figure 12:**
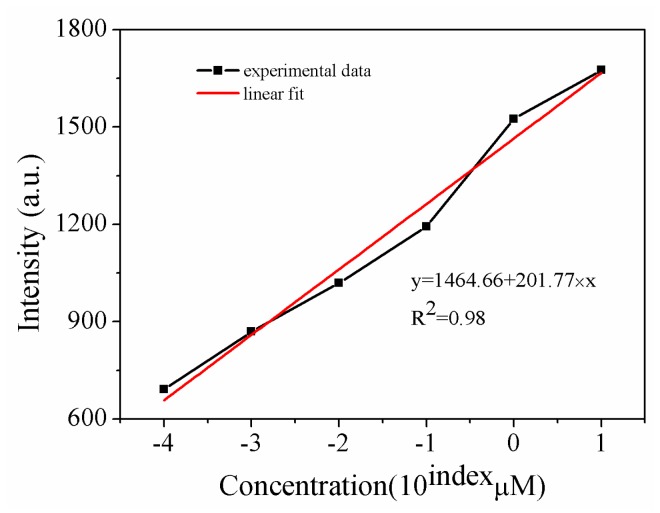
The fitting graph of the RRA-DNA concentration and fluorescence intensity.

THS-DNA (10 µM, 50 µL), which was connected with Au NPs/PSiMC substrate, causes hybridization under different concentrations (10 µM-10^−4^ µM, 40 µL) of complementary RRA-DNA. The fluorescence spectra are shown in Figure 11. There is a red shift for the emission peak of the fluorescence spectra with increasing DNA concentration. The reason for this phenomenon is that more DNA will attach to a gold nanoparticle with increasing DNA concentration, making the particle size increase and a red shift for the emission peak of the fluorescence spectra. According to the linear fitting graph (Figure 12), it shows a good linear correlation between the fluorescence intensity and the concentration, the minimum detectable concentration can reach 10^−5^ µM. Let us note that the sensitivity of the presented simple preparation and low cost assay is superior to that reported for complex biochip [31], enabling the detection of IgG molecules at the concention of 11 pM and Au nanograting [32] for the detection of DNA (100 pM). 

Although many studies based on SPR fluorescence enhancement for DNA detection have been carried out in the past few years [32,33,34], there remains a huge challenge of detecting short sequence DNA, because the short distance between Au NPs and fluorophore can hardly be useful for fluorescence enhancement. The demonstration of Au NPs-enhanced fluorescence for the detection of short sequence DNA in our sensing configuration will play an important role in designing future novel sensing platforms.

## 4. Conclusions

The fluorescence enhancement of probe RRA-DNA on the PSiMC substrate is better than that of the PSi Bragg reflector. The fluorescence intensity of RRA-DNA detected on the PSiMC substrate is twice as much as that on the Bragg reflector substrate. The results show that the fluorescence enhancement caused by plasmon resonance of Au NPs/PSiMC and Au NPs has advantages over that caused by the high reflectivity of the Bragg reflector. This fluorescence-enhanced substrate achieves highly sensitive DNA detection with a detection threshold of 10 pM. The enhancement of fluorescence emission on MC demonstrates that these structures are excellent materials to evolve into easy-to-use biosensors, which utilize the luminescence response of the molecules to be detected.

## Figures and Tables

**Table 1:** Experimental parameters of PSiMC.

Sample	Current Density	Corrosion Sequence
MC	Layer A: 110 mA/cm^2^	(AB)_6_AAAA(BA)_6_
	Layer B: 60 mA/cm^2^	
	Cavity layer: 110 mA/cm^2^	
Bragg reflector	Layer A: 110 mA/cm^2^	(AB)_14_
	Layer B: 60 mA/cm^2^	

**Table 2:** The sequence of DNA fragment.

Sample Number	DNA Fragment
Sample 1	Target DNA fragment: THS-5′-GGCCTATCAGCTTG-3′
	Probe1 DNA fragment: RRA-5′-CAAGCTGATAGGCC-3′
Sample 2	Target DNA fragment: THS-5′-GGCCTATCAGCTTG-3′
	Probe2 DNA fragment: RRA-5′-GGCCTATCAGCTTG-3′
Sample 3	Target DNA fragment: NH2-5′-GGCCTATCAGCTTG-3′
	Probe3 DNA fragment: RRA-5′-CAAGCTGATAGGCC-3′

## Supplementary Materials

The following are available online at http://www.mdpi.com/1424-8220/18/2/661/s1, Figure S1: (**a**) The zoom-in SEM figures of porous silicon microcavity (PSiMC), and (**b**) the hybrid nanostructure of gold nanoparticles (Au NPs) deposited on porous silicon.
