# Supplementary material for: Metal Nanoparticles/Porous Silicon Microcavity Enhanced Surface Plasmon Resonance Fluorescence for the Detection of DNA
Source: Sensors (Basel). 2018 Feb 23;18(2):661. doi: 10.3390/s18020661 (PMC5856129; doi:10.3390/s18020661)
Supplement: Supplementary file 1 [file sensors-18-00661-s001.pdf]

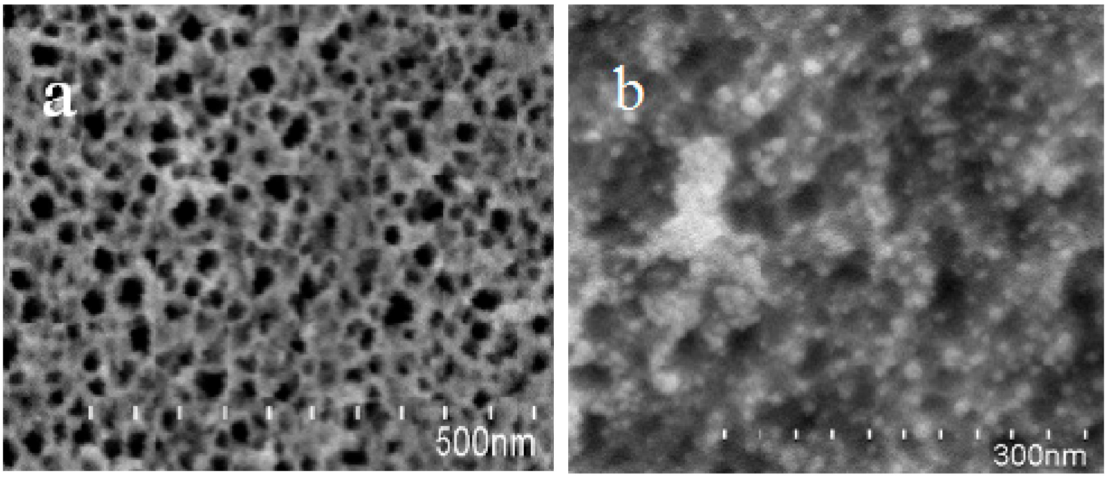

Figure. S1. (**a**) The zoom-in SEM figures of porous silicon microcavity (PSiMC), and (**b**) the hybrid nanostructure of gold nanoparticles (Au NPs) deposited on porous silicon.
